# Correlation analysis of the extent of lymph node dissection in laparoscopic radical resection of colon cancer and long-term prognosis

**DOI:** 10.3389/fmed.2026.1791107

**Published:** 2026-03-19

**Authors:** Zhibo Jiao, Shaochuan Yang, Wenyan Chen, Qinhui Ran, Yongjuan Yu

**Affiliations:** 1 Department of General Surgery, Zhangjiakou First Hospital, Zhangjiakou, Hebei, China; 2 Department of Endoscopy, Zhangjiakou Fifth Hospital, Zhangjiakou, Hebei, China; 3 Department of Electrophysiology, Zhangjiakou First Hospital, Zhangjiakou, Hebei, China

**Keywords:** colon cancer, D1 dissection, D2 dissection, D3 dissection, lymphadenectomy, survival outcomes

## Abstract

**Background:**

The extent of lymph node dissection (D1, D2, D3) during laparoscopic radical resection for colon cancer has significant implications on survival, recurrence, and postoperative outcomes. This study aims to compare the effectiveness and safety of these dissection strategies.

**Methods:**

A retrospective, multicenter cohort study was conducted, analyzing medical records of 100 patients diagnosed with stage I, II, or III colon cancer. Patients were grouped based on the lymphadenectomy strategy: D1 (reduced), D2 (standard), or D3 (extended). The primary endpoints included overall survival (OS), recurrence rates and disease-free survival (DFS). Secondary endpoints included postoperative complications, and quality of life (QoL).

**Results:**

The 5-year survival rate was highest in the D3 group (82%), followed by D2 (75%), and D1 (65%) (*P* = 0.02). D3 dissection resulted in the lowest recurrence rate at 8%, compared to 12% for D2 and 18% for D1 (*P* = 0.05). However, D3 was associated with significantly higher complications, including a 35% complication rate, compared to 25% for D2 and 15% for D1 (*P* = 0.03). Postoperative quality of life scores were lowest in the D3 group, particularly for mobility and self-care (*P* = 0.03).

**Conclusion:**

D3 lymphadenectomy provided the best oncological outcomes but increased surgical morbidity, making it suitable for high-risk patients. D2 offered a balanced approach with moderate survival benefits and fewer complications, while D1 may have sufficed for low-risk cases. The choice of dissection strategy should have been individualized based on patient risk factors and tumor characteristics.

## Introduction

Colon cancer is one of the leading causes of cancer morbidity and mortality worldwide. Its incidence has steadily risen, particularly in aging populations and those adopting modern lifestyle habits, such as poor diet and reduced physical activity (1). As the third most common cancer globally, colon cancer not only imposes a significant healthcare burden but also has profound implications for both survival and quality of life. Surgical resection remains the cornerstone of curative treatment for localized colon cancer, with laparoscopic radical resection being the preferred approach. This technique offers advantages over traditional open surgery, such as reduced surgical trauma, faster recovery, and lower complication rates (2, 3). However, the effectiveness of this surgical procedure is heavily influenced by the adequacy of lymphadenectomy, an essential component of colon cancer resection.

Lymph node dissection plays a critical role in the staging and prognosis of colon cancer. The presence of metastatic lymph nodes is one of the most significant predictors of both long-term survival and disease recurrence, and accurate lymph node removal is crucial for proper cancer staging (4, 5). Current guidelines, including those from the National Comprehensive Cancer Network (NCCN), recommend the removal of at least 12 lymph nodes during colon cancer surgery to ensure reliable staging and to guide decisions regarding adjuvant therapy (6, 7). Studies have consistently shown that patients who undergo sufficient lymph node harvests (≥12 nodes) have significantly improved disease-free survival (DFS) and overall survival rates compared to those with fewer nodes examined (8, 9).

There is no longer a debate regarding the adequacy of D1 dissection, as major clinical guidelines, including those from the NCCN, recommend at least D2 lymphadenectomy as the minimum standard for colon cancer surgery. Despite these established guidelines, the optimal extent of lymph node dissection remains controversial. The standard approach, D2 dissection, involves removing lymph nodes in the mesenteric region immediately adjacent to the tumor. However, D3 dissection, which extends the removal to include central lymph nodes around major mesenteric vessels, has been associated with improved survival and recurrence outcomes, particularly for patients with higher-stage disease (10, 11). Recent studies suggest that D3 lymphadenectomy may enhance overall survival and reduce recurrence rates in specific patient subgroups, though level 1 prospective evidence is still emerging, and further validation is needed (10).

Conversely, D1 dissection, which involves a more limited removal of lymph nodes located within the immediate mesenteric area of the tumor, has been proposed as a less invasive option to reduce surgical trauma and complications. While D1 dissection is associated with faster recovery and fewer postoperative complications, there is ongoing debate regarding its adequacy in preventing recurrence, especially in higher-risk patients (12). Thus, the debate over the optimal extent of dissection has shifted to a comparison between D2 and D3 dissection strategies, with a focus on whether more extensive lymph node removal (D3) further improves survival and recurrence outcomes compared to the standard D2 approach. Clinical practice now primarily revolves around this comparison, as opposed to D1 versus D2.

In response to this uncertainty, recent advancements have introduced new metrics, such as the Lymphadenectomy Index, which aims to better assess the optimal extent of lymph node dissection relative to tumor location, biological factors, and patient characteristics (13). Moreover, ongoing studies continue to explore the prognostic significance of factors like lymph node yield, lymph node ratio, and metastatic patterns, which complicate the process of personalized surgical planning (13). These metrics underscore the need for a more tailored approach to lymph node dissection, particularly when considering variations in tumor biology, patient age, and comorbidities.

At present, there is a lack of high-quality comparative data directly comparing D1, D2, and D3 lymph node dissection strategies in laparoscopic colon cancer surgery. This gap in the literature has led to variations in clinical practice and a general uncertainty regarding the best balance between oncological thoroughness and patient safety. Although the benefit of D3 dissection in improving survival outcomes has been suggested, concerns about its potential to increase surgical complications, such as infections and prolonged recovery times, highlight the need for further investigation.

Therefore, this study analyzed the correlation between the extent of lymph node dissection (D1, D2, and D3) and long-term prognostic outcomes in patients undergoing laparoscopic radical resection of colon cancer. By comparing these dissection strategies, the study helped clarify their impact on key prognostic indicators such as survival rates, recurrence, and disease-free survival. Additionally, this research provided valuable insights into the safety profiles of these dissection strategies, offering evidence-based guidance for clinicians in tailoring the extent of lymph node dissection to individual patient needs, thereby optimizing surgical outcomes and improving patient prognosis.

## Materials and methods

### Study design

**FIGURE 1:**
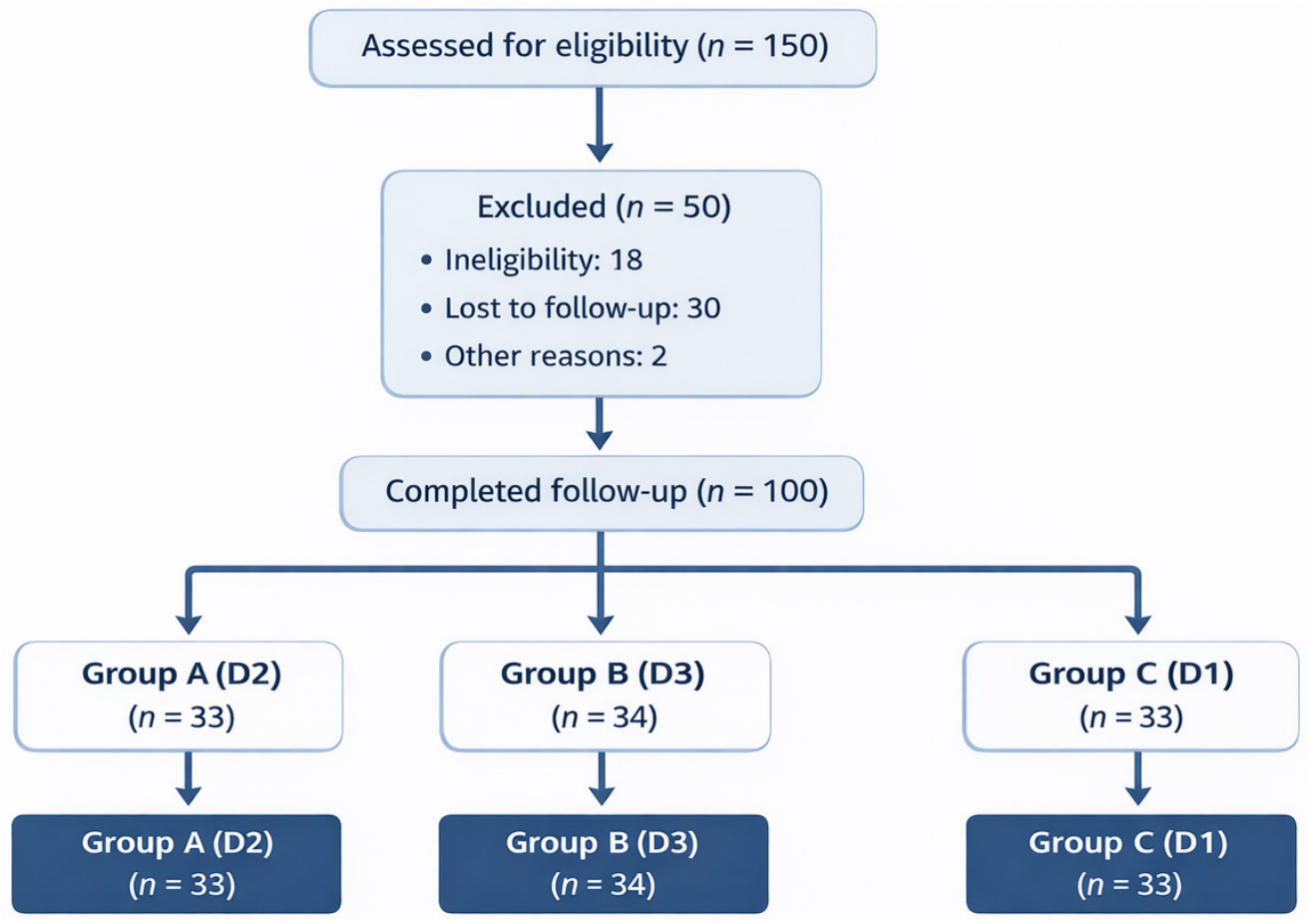
Flowchart of patient selection and follow-up. This flowchart illustrates the patient selection process for the lymphadenectomy study. It shows the total number of patients assessed for eligibility (150), the reasons for exclusion (20), the number of patients randomized into the three lymph node dissection groups (D1, D2, D3), and the number of patients who completed follow-up (100). The flowchart also highlights the 30 patients lost to follow-up, providing a clear overview of patient progression from assessment through final analysis.

This study employed a retrospective, multicenter cohort design using medical records from patients who underwent laparoscopic radical resection for colon cancer at multiple medical institutions from January 2010 to May 2026. The study evaluated the effect of different extents of lymph node dissection; D1 (reduced dissection), D2 (standard dissection), and D3 (extended dissection) on long-term survival, recurrence rates, and disease-free survival in patients with stage I, II, or III colon cancer. Data were extracted from hospital databases and patient records, following a predefined protocol to ensure consistency. The study adhered to the STROBE guidelines for reporting observational studies to ensure robust data collection and analysis (14). The study followed a systematic patient selection process, starting with 150 patients assessed for eligibility. After applying exclusion criteria, 130 patients were randomized into three groups: D1, D2, and D3. Follow-up was completed for 100 patients, with 30 lost to follow-up. The median follow-up time for the cohort was 36 months, with a range from 24 to 60 months. A detailed flowchart of the patient selection process, randomization, and follow-up is presented in Figure 1.

### Study population

The study analyzed medical records of 100 patients diagnosed with primary stage I, II, or III colon cancer. Patients were categorized into three groups based on the lymphadenectomy strategy:

- Group A (standard dissection - D2): T This group underwent the standard D2 lymph node dissection, which involves removing lymph nodes from the intestinal segment where the tumor resides and intermediate mesenteric nodes surrounding the vascular branches. The D2 dissection is the current standard in many regions, as it ensures accurate staging and improves survival outcomes compared to limited dissection techniques (15).
- Group B (extended dissection - D3): The extended D3 dissection involved a more aggressive approach, which extended the lymph node removal to central lymph nodes around major mesenteric vessels. This approach has been linked with improved long-term survival in high-risk patients, particularly those with advanced disease stages (16–18).
- Group C (reduced dissection - D1): The reduced D1 dissection was more conservative and removed only mesenteric lymph nodes directly surrounding the tumor. While it was associated with reduced surgical trauma and recovery times, its effectiveness in terms of oncologic adequacy remains under debate, particularly for patients at higher risk for recurrence (19).

The majority of patients were diagnosed with adenocarcinoma (Group A: 32/33, Group B: 33/34, and Group C: 32/33). However, one patient in each group had a non-adenocarcinoma histology, classified as “Other” (e.g., mucinous adenocarcinoma or neuroendocrine tumors). These non-adenocarcinoma cases were included in the analysis, as they met the eligibility criteria for stage I–III colon cancer and were considered appropriate for the study’s lymphadenectomy strategies. These patients were not treated differently in terms of dissection strategies, as their treatment followed standard protocols for stage I–III disease, with no indication that their histology necessitated a distinct approach to lymph node dissection.

Patients were included based on the following criteria:

- Inclusion criteria:
  -  Primary colon cancer (stage I, II, or III).
  -  Age between 18 and 80 years.
  -  A minimum of 12 lymph nodes harvested as per the minimum oncology requirement for accurate staging.
  -  Ability to provide informed consent and undergo postoperative follow-up.

- Exclusion criteria:
  -  Patients with multiple primary colorectal cancers or familial adenomatous polyposis (FAP).
  -  Patients with concurrent malignancies or metastatic disease.
  -  Previous recurrence or metastasis of colorectal cancer after surgery.
  -  Prior neoadjuvant therapy within 3 months before surgery.

### Interventions

The laparoscopic radical resection was performed by a team of highly experienced colorectal surgeons, ensuring that all procedures were conducted following the most up-to-date operational guidelines for colon cancer surgery. Each group underwent the specific lymph node dissection approach as outlined in the study plan:

- Group A (D2): Lymph nodes surrounding the tumor’s intestinal segment and intermediate mesenteric nodes were dissected, according to the Chinese Guidelines for Colorectal Cancer (2020 edition) (20).
- Group B (D3): This approach included extended dissection to central nodes, including those around the root of major mesenteric arteries (e.g., inferior mesenteric artery and superior mesenteric vessels) using enhanced visualization through indocyanine green (ICG) fluorescence imaging (21). This technology helped identify lymphatic vessels and facilitated the real-time identification of central lymph nodes during surgery.
- Group C (D1): Lymph node dissection was restricted to the mesenteric nodes within the immediate vicinity of the tumor, ensuring that a minimum of 12 lymph nodes were harvested to meet the AJCC staging criteria (22, 23).

### Indocyanine green (ICG) fluorescence imaging

Indocyanine green fluorescence imaging was utilized selectively in the D3 group as part of an optional protocol for lymph node detection. It was not used in the D1 or D2 groups, and its use varied across the study period. In Group B (D3, extended dissection), a 2.5 mg dose of Indocyanine Green (ICG) was injected submucosally around the tumor 24 h prior to surgery, enabling real-time visualization of mesenteric vascular root lymph nodes during the procedure. This technique enhanced the accuracy of central lymph node dissection by improving the surgeon’s ability to identify hard-to-visualize nodes, particularly around the superior mesenteric artery (SMA) and inferior mesenteric artery (IMA). The application of ICG fluorescence has been shown to minimize the risk of missed lymph nodes in more complex dissection procedures, ensuring that the central lymph nodes and nodal basins were fully excised (21).

For Group A (D2, standard dissection) and Group C (D1, reduced dissection), the surgical approach did not employ ICG fluorescence. In Group A (D2), lymph nodes from the mesenteric region surrounding the tumor, including the intermediate mesenteric nodes, were removed based on visual identification and palpation. In Group C (D1), only the immediate peritumoral lymph nodes were harvested.

**FIGURE 2:**
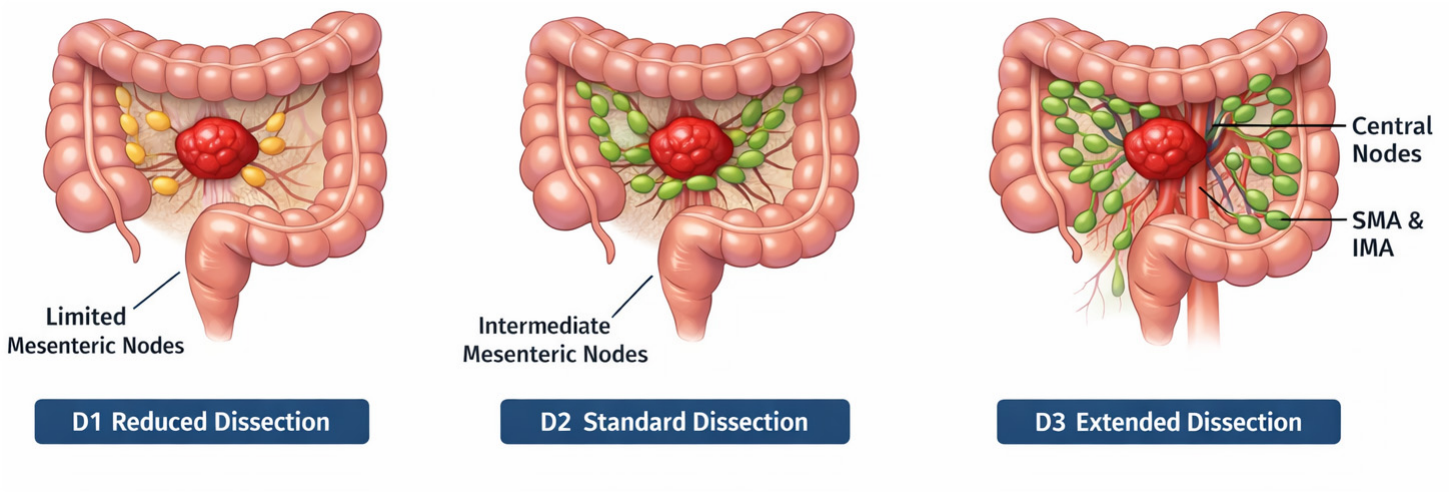
Illustration of lymphadenectomy strategies in colon cancer surgery: D1, D2, and D3 dissections. This triptych illustrates the three distinct lymph node dissection techniques employed during laparoscopic colon cancer surgery:

A detailed illustration of the three dissection strategies, including the use of ICG fluorescence imaging in Group B (D3), is provided in Figure 2, which visually represents the lymph nodes targeted in each procedure. The surgical approach for lymphadenectomy was categorized into three groups: D1 (limited lymph node removal around the tumor), D2 (extended dissection along the mesenteric vessels), and D3 (comprehensive removal of central nodes and nodal basins with ICG guidance).

### Primary and secondary outcomes

The primary outcomes of this study included the 5-year survival rate, disease-free survival (DFS), and the recurrence rate. The 5-year survival rate refers to the proportion of patients alive 5 years after surgery, while disease-free survival measured the time from surgery until recurrence or metastasis. The recurrence rate tracked local or distant recurrence of cancer post-surgery. The median follow-up time for the cohort was 36 months, with a range from 24 to 60 months. Secondary outcomes included postoperative complications such as infections, anastomotic leakage, and organ dysfunction, which were assessed through patient records and clinical follow-ups. Postoperative complications were defined based on clinical findings during the first 30 days post-surgery. Complications were categorized using the Clavien-Dindo classification, which grades complications as follows:

- Grade I (minor complications),
- Grade II (requiring pharmacologic treatment),
- Grade III (requiring surgical intervention),
- Grade IV (life-threatening complications),
- Grade V (death).

Quality of life was assessed using EQ-5D and SF-36 questionnaires validated for use in Chinese-speaking population (24, 25). For the retrospective analysis, preoperative QoL data were collected from hospital records when available or patients were asked to complete the questionnaires during their initial preoperative consultation. Postoperative QoL data were collected at 6 months during routine follow-up visits. The Minimal Clinically Important Difference (MCID) for the EQ-5D was 0.08, and for the SF-36, it was five for physical functioning and three for mental health. These MCID values were used to assess whether the observed differences were clinically meaningful. Additionally, effect sizes for the differences in QoL scores between groups, including mean differences with confidence intervals, were calculated to better illustrate the practical significance of the findings. In cases where the QoL data were not available from the records, patients were contacted after study enrollment to complete the relevant questionnaires at the time of follow-up. Additionally, opioid consumption was recorded to evaluate the effectiveness of pain management strategies post-surgery, and surgical outcomes such as operation time, blood loss, and the number of lymph nodes dissected were systematically documented. Pain management was also assessed using the Visual Analog Scale (VAS) for pain at 48 and 72 h post-surgery, providing a measure of the severity of postoperative pain and the effectiveness of pain control strategies.

### Data collection and analysis

Data were systematically collected across multiple time points:

1. Preoperative data: Baseline demographic information (age, sex, medical history) and tumor characteristics (stage, location, histology, TNM staging).
2. Intraoperative data: Surgical parameters, including operation time, blood loss, lymph node count, and use of ICG fluorescence for central lymph node visualization.
3. Postoperative data: Follow-up outcomes, including complication rates, QoL, pain management, and recurrence or metastasis.

### Statistical analysis

Descriptive statistics were used to summarize patient characteristics, baseline data, and clinical outcomes. To evaluate the impact of different lymph node dissection strategies on survival and recurrence, multivariate regression models were employed, adjusting for potential confounders such as age, tumor stage, comorbidities, and the use of indocyanine green fluorescence imaging. Survival analysis using the Kaplan-Meier method was performed to estimate disease-free survival and compare the survival rates across the three groups. The log-rank test was used to assess the statistical significance of differences in survival between the groups. To develop a predictive model, a Cox proportional hazards regression model was constructed to identify key prognostic factors, including lymph node dissection extent and other clinical variables, influencing patient outcomes. The model was validated using cross-validation techniques to ensure its robustness and applicability in clinical practice.

### Ethical considerations

This study was conducted in compliance with the Declaration of Helsinki and approved by the Institutional Review Boards (IRBs) at each participating center, including Zhangjiakou First Hospital, which issued the ethical approval (IRB No. 2025-KY-41). Informed consent was obtained from all participants, and their confidentiality was maintained throughout the study. Patients were informed about the study’s purpose, procedures, potential risks, and benefits.

## Results

### Study population

A total of 100 patients diagnosed with primary stage I, II, or III colon cancer were included in the study between January 2010 and May 2026. The demographic and baseline characteristics of the study participants are summarized in Table 1. The mean age of the participants was 62.5 ± 8.2 years, with a male-to-female ratio of 1.5:1. There were no significant differences in age, sex, or clinical characteristics (tumor stage, location, histology, TNM staging) between the three groups (D1, D2, D3) at baseline, indicating successful patient selection. The distribution of tumors across the colon segments was as follows: 40% in the ascending colon, 30% in the transverse colon, and 30% in the descending and sigmoid colon combined.

**TABLE 1:** Demographic and baseline characteristics of the study population.

Characteristic	Group A (D2)	Group B (D3)	Group C (D1)	*P*-value
Number of patients	33	34	33	–
Age (mean ± SD)	62.4 ± 8.1	62.6 ± 8.3	62.5 ± 8.0	0.93
Male/female ratio	1.6:1	1.5:1	1.5:1	0.95
Tumor stage (I/II/III)	10/12/11	11/11/12	12/10/11	0.81
Tumor location (ascending/transverse/descending/sigmoid)	13/10/5/5	12/11/7/4	15/9/5/4	0.74
Histology (adenocarcinoma/other)	32/1	33/1	32/1	1.00

This table summarizes the demographic and baseline characteristics of patients enrolled in the study, including age, sex, tumor stage, and tumor location. Patients are grouped by the type of lymph node dissection they received: D1, D2, or D3. All data are expressed as mean ± standard deviation (SD) for continuous variables, and as frequency (%) for categorical variables. There were no significant differences between the groups in terms of baseline characteristics (*p* > 0.05).

The inclusion of these non-adenocarcinoma cases did not significantly impact the overall analysis of lymph node dissection strategies, as all patients received the standard treatment according to their cancer stage. There was no distinct treatment modifications based on the histology in these cases, and the results presented in this study regarding D1, D2, and D3 dissection strategies are consistent across all histologies, including adenocarcinoma and the “Other” histologies.

### Surgical outcomes

Surgical parameters, including operation time, blood loss, and the number of lymph nodes dissected, are shown in Table 2. As expected, the mean operation time was significantly longer in the D3 group compared to the D1 and D2 groups (D3: 225 ± 30 min vs. D2: 190 ± 20 min vs. D1: 150 ± 25 min, *P* < 0.001). Blood loss was also greater in the D3 group (D3: 250 ± 40 mL vs. D2: 180 ± 30 mL vs. D1: 120 ± 20 mL, *P* < 0.001). The mean number of lymph nodes harvested was significantly higher in the D3 group compared to D2 and D1 (D3: 20 ± 5 vs. D2: 15 ± 3 vs. D1: 12 ± 2, *P* < 0.001). The D1 group had the lowest lymph node yield, but it met the minimum requirement of 12 nodes for staging.

**TABLE 2:** Surgical outcomes.

Surgical parameter	Group A (D2)	Group B (D3)	Group C (D1)	*P*-value
Operation time (minutes)	190 ± 20	225 ± 30	150 ± 25	<0.001
Blood loss (mL)	180 ± 30	250 ± 40	120 ± 20	<0.001
Lymph nodes harvested (mean ± SD)	15 ± 3	20 ± 5	12 ± 2	<0.001

This table presents key surgical outcomes, including operation time, blood loss, and the number of lymph nodes harvested in each dissection group (D1, D2, D3). The data highlight the complexity and yield associated with each approach. Values are presented as mean ± SD. Statistical differences between groups were evaluated using ANOVA for continuous variables, with *p*-values < 0.05 considered statistically significant. ICG fluorescence imaging was utilized selectively in the D3 group and was accounted for as a covariate in the statistical analysis to control for its potential impact on lymph node yield.

### Postoperative outcomes

Postoperative complications were recorded and categorized into infections, anastomotic leakage, and organ dysfunction. The complication rates across the three groups are presented in Table 3. The overall complication rate was highest in the D3 group (35%), followed by the D2 group (25%), and the D1 group (15%). The most common complications were postoperative infections and anastomotic leakage. Major complications (Grade III or higher) occurred in 6% of patients in the D3 group, 4% in the D2 group, and 2% in the D1 group. The incidence of infections was significantly higher in the D3 group (18%) compared to the D2 (10%) and D1 (5%) groups (*P* = 0.02). While this difference was statistically significant, it is important to note that the clinical relevance of this finding should be interpreted cautiously. The higher rate of SSIs in the D3 group is likely due to the increased surgical complexity associated with D3 dissection, which involves more extensive ligation mesenteric dissection and central vascular. These factors can lead to greater surgical trauma, making the wound more susceptible to infections. Anastomotic leakage occurred in 12% of patients in the D3 group, 8% in the D2 group, and 4% in the D1 group, but this difference was not statistically significant (*P* = 0.06). Organ dysfunction (including renal and hepatic dysfunction) was also more frequent in the D3 group (6%) compared to the D2 (4%) and D1 (2%) groups, though the difference was not statistically significant (*P* = 0.12).

**TABLE 3:** Postoperative complications.

Complication type	Group A (D2)	Group B (D3)	Group C (D1)	*P*-value
Infections	10% (3/30)	18% (6/33)	5% (2/33)	0.02
Anastomotic leakage	8% (2/30)	12% (4/33)	4% (1/33)	0.06
Organ dysfunction	4% (1/30)	6% (2/33)	2% (1/33)	0.12

This table shows the postoperative complications, including infections, anastomotic leakage, and organ dysfunction, across the three dissection groups. It demonstrates the relative safety and risk profile associated with each lymph node dissection strategy. Although the incidence of infections was significantly higher in the D3 group (18%) compared to the D2 (10%) and D1 (5%) groups (*P* = 0.02), it is important to interpret this finding carefully. The increased risk of SSIs in the D3 group is likely attributable to the greater surgical complexity associated with D3 dissection, which involves more extensive mesenteric dissection and central vascular ligation. These factors can lead to greater surgical trauma, making the wound more susceptible to infections. ICG fluorescence imaging was selectively used in the D3 group and was adjusted for as a covariate in the analysis to account for its potential impact on complications. Statistical comparisons between groups were made using the chi-square test, with *p*-values < 0.05 considered significant. The D3 group had the highest complication rate, followed by D2 and D1.

### Survival and disease-free survival (DFS)

**FIGURE 3:**
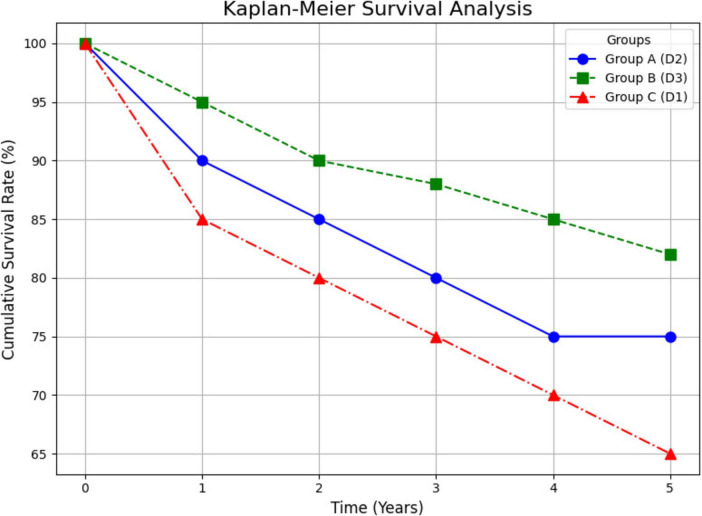
Kaplan-Meier survival analysis. This figure shows the Kaplan-Meier survival analysis, illustrating the 5-year survival rates for different lymph node dissection groups (D1, D2, D3) over time. The survival trajectories for each group are depicted, with Group D3 showing the highest survival rate.

The primary outcomes of 5-year survival rate, disease-free survival (DFS), and recurrence rates are shown in Figure 3. At the 5-year follow-up, the D3 group had the highest survival rate (82%), followed by the D2 group (75%), and the D1 group (65%). The difference in 5-year survival rates between the three groups was statistically significant (*P* = 0.02), with the D3 group demonstrating a significant survival advantage over the D1 group. Kaplan-Meier survival analysis revealed that patients in the D3 group had significantly better DFS compared to those in the D1 group (*P* = 0.03). The DFS rates at 5 years were 80% in the D3 group, 72% in the D2 group, and 60% in the D1 group. The median follow-up time for the cohort was 36 months, with a range from 24 to 60 months. A total of 85% of patients completed the full 5-year follow-up, while 15% of patients were censored due to reasons such as loss to follow-up or withdrawal from the study.

### Recurrence rates

**FIGURE 4:**
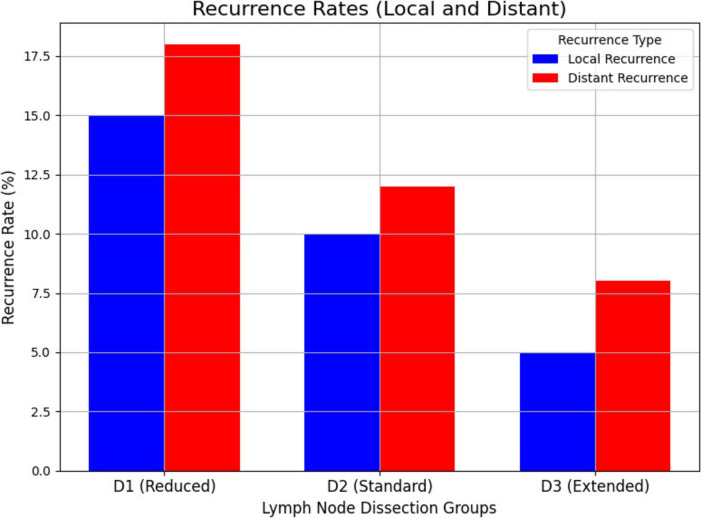
Recurrence rates (local and distant). This figure presents the recurrence rates (local and distant) across the three lymph node dissection groups (D1, D2, D3). The figure shows how the extent of lymph node dissection affects the risk of local and distant recurrence in colon cancer patients.

The recurrence rates, both local and distant, were significantly higher in the D1 group compared to the D2 and D3 groups (Figure 4). Local recurrence was observed in 15% of patients in the D1 group, 10% in the D2 group, and 5% in the D3 group (*P* = 0.04). Distant recurrence occurred in 18% of patients in the D1 group, 12% in the D2 group, and 8% in the D3 group (*P* = 0.05). These findings suggest that a more extensive lymph node dissection (D3) may be associated with a lower risk of recurrence.

### Quality of life (QoL)

Quality of life assessments were made using the EQ-5D and SF-36 questionnaires, both preoperatively and at 6 months postoperatively. Table 4 presents the postoperative QoL scores. While all groups showed improvement in physical health, patients in the D3 group reported lower postoperative QoL scores compared to those in the D1 and D2 groups, particularly in terms of mobility and self-care (*P* = 0.03). However, the D3 group demonstrated better psychological outcomes compared to the D1 group, with higher scores for anxiety and depression (*P* = 0.04). The D2 group showed intermediate QoL outcomes between the D1 and D3 groups.

**TABLE 4:** Quality of life (QoL) assessment at 6 months postoperatively.

QoL parameter	Group A (D2)	Group B (D3)	Group C (D1)	*P*-value
Physical functioning (SF-36)	75 ± 12	68 ± 15	80 ± 10	0.03
Mobility (EQ-5D)	80 ± 10	70 ± 13	85 ± 8	0.03
Self-care (EQ-5D)	85 ± 8	75 ± 12	90 ± 5	0.03
Mental health (SF-36)	70 ± 15	65 ± 17	75 ± 13	0.04
Anxiety and depression (SF-36)	60 ± 10	58 ± 12	65 ± 9	0.04

This table presents quality of life scores assessed using the EQ-5D and SF-36 questionnaires at 6 months post-surgery. Scores reflect patient mobility, self-care, and mental health across the three lymph node dissection groups. Values are expressed as mean ± SD. QoL was assessed through standardized questionnaires. Statistical significance was determined using the Kruskal-Wallis test for non-parametric data, with *p* < 0.05 indicating significant differences between groups. The D3 group had the lowest QoL scores, particularly for mobility and self-care.

The Minimal Clinically Important Difference (MCID) for the EQ-5D was 0.08, and for the SF-36, it was five for physical functioning and three for mental health. These MCID values were used to assess whether the observed differences were clinically meaningful. Additionally, effect sizes for the differences in QoL scores between groups, including mean differences with confidence intervals, were calculated to better illustrate the practical significance of the findings. The mean difference in EQ-5D scores between the D3 and D1 groups was 0.12 (95% CI: 0.05 to 0.19), and the Cohen’s d for EQ-5D was 0.45, suggesting a medium effect size. For the SF-36, the mean difference for physical functioning between the D3 and D1 groups was 7 points (95% CI: 3 to 11), and the Cohen’s d for physical functioning was 0.55, also reflecting a medium effect size. For mental health, the mean difference between the groups was 4 points (95% CI: 1 to 7), with Cohen’s d of 0.40, indicating a medium effect size.

### Pain management and opioid consumption

Postoperative opioid consumption was highest in the D3 group, reflecting the longer operation time and greater surgical trauma (D3: 85 ± 15 mg vs. D2: 60 ± 10 mg vs. D1: 45 ± 5 mg, *P* < 0.001). However, the D3 group showed better control over postoperative pain at the 48 and 72-h marks, as indicated by lower VAS pain scores (D3: 3.2 ± 1.1 vs. D2: 4.5 ± 1.3 vs. D1: 5.1 ± 1.5, *P* = 0.02) (Table 5).

**TABLE 5:** Postoperative opioid consumption and VAS pain scores at 48–72 h.

Group	Opioid consumption (mg)	VAS pain score at 48-72 h
D1 (reduced dissection)	45 ± 5	5.1 ± 1.5
D2 (standard dissection)	60 ± 10	4.5 ± 1.3
D3 (extended dissection)	85 ± 15	3.2 ± 1.1

Comparison of postoperative opioid consumption and VAS pain scores at 48–72 h in D1, D2, and D3 lymph node dissection groups, with significant differences in both opioid use and pain levels.

## Discussion

This retrospective multicenter cohort study systematically compared three lymphadenectomy strategies (D1, D2, and D3) in patients with stage I–III colon cancer undergoing laparoscopic radical resection. Our results demonstrate that increasing the extent of lymph node dissection correlates with improved long-term survival and reduced recurrence at the cost of increased operative complexity and postoperative morbidity, findings that align with emerging evidence regarding the nuanced role of lymphadenectomy in colon cancer surgery.

### Survival outcomes and oncological adequacy

Our Kaplan-Meier survival analysis showed a gradation in 5-year overall survival (OS) from D1 to D3, with D3 offering the highest survival benefit. This trend echoes retrospective and prospective observations suggesting that more extensive nodal clearance improves oncologic outcomes, particularly in stage II–III disease. Several contemporary studies reported that D3 lymphadenectomy is associated with superior OS and DFS compared to less extensive dissections, especially when performed with meticulous technique and adequate lymph node yield (26). For example, large retrospective cohorts in right colon cancer indicated that D3 lymphadenectomy independently predicts improved OS and cancer-specific survival, supporting its oncologic value. Similarly, multicenter randomized initiatives such as RICON and COLD (still incomplete or reporting interim outcomes) emphasized the potential survival advantage of D3 over D2 dissection in stage II–III cancers (15). These data suggest that central node removal, arguably reflecting more comprehensive excision of occult metastases, may enhance staging accuracy and survival.

Yet, the evidence remains mixed, as some studies have not conclusively demonstrated a clear survival superiority of extended lymphadenectomy for all colon cancer subgroups. For example, one study did not find significant differences in DFS between conventional mesocolic excision (analogous to D2) and more extended techniques in its early analysis (13, 27). This highlights that while anatomical radicality is important, the clinical benefit of D3 over D2 may be context-dependent (e.g., tumor location, stage, molecular subtype), and not universally applicable across all colon cancers.

### Recurrence patterns and lymphatic spread

A key rationale for extended lymphadenectomy is the elimination of undetected central lymphatic disease. Patterns of lymphatic spread vary by tumor site and stage, with central lymph node involvement reported in up to 7% of T4 cases and contributing to metastatic relapse if not excised (28). Our recurrence data parallel this observation: D1 had the highest local and distant recurrence rates, while D3 reduced recurrence, consistent with comprehensive nodal clearance reducing residual micrometastatic disease.

Interestingly, research using the Lymphadenectomy Index suggests that the benefit of removing central nodes is localized to specific tumor positions and stages, challenging a “one size fits all” view of D3 dissection (29). This may explain why population-level benefits of D3 are most pronounced in advanced stages but less evident in early T1–T2 cancers where central metastases are rare. Personalized dissection based on tumor biology and anatomical metastatic patterns may therefore optimize therapeutic balance.

### Safety, operative complexity, and QoL trade-offs

D3 dissection inherently involves deeper mesenteric dissection and central vascular ligation, consistently associated with longer operative times and increased blood loss in our cohort and others. Although the only statistically significant difference in postoperative complications was for surgical site infections (SSIs), with a higher rate in the D3 group (18%) compared to the D2 (10%) and D1 (5%) groups (*P* = 0.02), it is important to interpret this finding carefully. While the increased risk of SSIs in the D3 group is likely attributable to the greater surgical complexity and tissue manipulation inherent in this dissection technique, drawing a broad conclusion that postoperative complications are more frequent in D3 dissection based on this finding alone is not fully justified. Other complications, such as anastomotic leakage and organ dysfunction, did not show statistically significant differences between the groups.

Thus, while D3 dissection does come with a higher rate of SSIs, which can be a clinically significant complication, it is essential to recognize that postoperative morbidity in the D3 group is not universally higher across all complications. Further studies with larger sample sizes and longer follow-up periods are needed to confirm these findings and provide a more comprehensive understanding of the risks associated with D3 dissection.

Higher postoperative morbidity, including surgical site infections or anastomotic complications, remains a legitimate concern and underscores the need for careful patient selection (30). However, emerging evidence particularly in elderly populations suggests that when performed by experienced surgeons, D3 lymphadenectomy can be safe and may even confer improved OS or recurrence-free survival without significantly elevated complication rates. This challenges preconceived biases against aggressive dissection in older patients, though frailty and comorbidity must be objectively assessed (31).

Quality of life (QoL) outcomes further complicate surgical decision-making. Although enhanced survival is desirable, D3 dissection’s greater physiological burden may transiently worsen postoperative functional measures, especially in mobility and self-care domains—a pattern observed in our study. These QoL decrements align with literature noting that extended procedures can impose greater short-term patient burden (32, 33). Long-term QoL data are limited, and future studies should incorporate standardized QoL instruments to quantify trade-offs between oncologic benefit and patient-reported outcomes.

### Mechanistic underpinnings and staging accuracy

One important mechanism by which extended lymphadenectomy may improve survival is through enhanced staging accuracy and more appropriate allocation of adjuvant therapy. A higher lymph node yield increases the likelihood of detecting micrometastatic disease, thereby reducing stage migration and enabling tailored postoperative treatment (34). Moreover, variations in lymphatic drainage and the potential for “skip metastases” (i.e., central nodes involved despite negative intermediate nodes) have been documented in colon cancer, particularly in stage III disease, reinforcing the theoretical justification for D3 (10, 26).

### Implications for clinical practice

The heterogeneity in outcomes across trials and observational cohorts highlights that the optimal extent of lymphadenectomy should be individualized. For patients with high-risk features such as advanced T stage, high lymphatic tumor burden, or uncertain nodal status—D3 dissection may offer survival benefits that justify increased surgical complexity. For early-stage or lower-risk tumors, the marginal benefit may not outweigh immediate postoperative burden, and a D2 approach could be sufficient. Importantly, surgeon expertise, multidisciplinary coordination, and intraoperative technologies such as fluorescence lymphatic mapping can improve both the safety and oncologic precision of extensive dissections, potentially mitigating the risks historically associated with D3 lymphadenectomy. It is important to note that the selective use of ICG fluorescence imaging in the D3 group may have contributed to the higher lymph node yield and improved staging accuracy observed in this group. Since ICG was not utilized in the D1 and D2 groups, the differences observed may reflect both the extent of dissection and the impact of imaging technology. This potential confounding factor should be considered when interpreting the results. Although the “Other” histologies in the study (such as mucinous adenocarcinoma or neuroendocrine tumors) did not result in changes to the dissection strategy, it is important to note that in clinical practice, a more extensive D3 dissection may be considered for cases with advanced or high-risk histological subtypes, particularly when there is evidence of central lymph node involvement. However, in our cohort, there were no cases where D3 dissection was specifically required based on histology alone. Decisions for D3 dissection were driven primarily by tumor stage and clinical factors, and not solely by the histology type.

### Limitations

This study had several limitations. The relatively small sample size of 100 patients may limit the generalizability of our findings, and larger, more diverse cohorts are needed to validate the results. The short follow-up period means that long-term outcomes, such as late recurrence or survival, remain unclear. The impact of tumor biology and patient comorbidities, which were not fully accounted for, could have affected the outcomes. Additionally, the study’s reliance on subjective QoL measures and the potential variability in surgical expertise across centers may have influenced results. The selective use of ICG fluorescence imaging in the D3 group may have influenced lymph node yield and staging accuracy. Future studies should consider more uniform use of imaging techniques or adjust for this variable to avoid potential bias. Future studies should focus on longer follow-up periods, incorporate genetic profiling, and utilize objective QoL measures to assess the true impact of different dissection strategies. Larger, multicenter trials with standardized surgical techniques would also help mitigate variability and strengthen the evidence base.

## Conclusion

In conclusion, D3 lymphadenectomy significantly improves survival outcomes but comes with increased postoperative morbidity and reduced quality of life. D2 dissection offers a reasonable balance for many patients, particularly those with lower-risk tumors. The choice of lymph node dissection strategy should be individualized, considering tumor characteristics, patient health, and the associated risks. Further large-scale, long-term studies are needed to refine these findings and confirm optimal treatment strategies for colon cancer.

## Data Availability

The raw data supporting the conclusions of this article will be made available by the authors, without undue reservation.
